# MICAL1 Contributes to Myogenic Differentiation by Modulating Actin Remodeling and YAP1 Nuclear Localization in C2C12 Myoblasts

**DOI:** 10.3390/ijms27146505

**Published:** 2026-07-22

**Authors:** Thanh Huu Phan Ngo, Quoc Kiet Ly, Wan Lee

**Affiliations:** 1 Department of Biochemistry, Dongguk University College of Medicine, 123 Dongdae-ro, Gyeongju 38066, Republic of Korea; thngo139@gmail.com (T.H.P.N.); kietly1501@gmail.com (Q.K.L.); 2 Section of Molecular and Cellular Medicine, Medical Institute of Dongguk University, Dongguk University College of Medicine, 123 Dongdae-ro, Gyeongju 38066, Republic of Korea

**Keywords:** MICAL1, flavoprotein monooxygenase, actin remodeling, YAP1, mechanotransduction, myogenic differentiation, cell cycle

## Abstract

Molecule Interacting with CasL 1 (MICAL1) is a flavoprotein monooxygenase that promotes filamentous actin (F-actin) depolymerization. Transcriptomic studies have linked MICAL1 downregulation to skeletal muscle atrophy and muscular dystrophy, yet its functional contribution to myogenesis remains unexplored. We found that MICAL1 protein increased progressively during myogenic differentiation of C2C12 cells, reaching a maximum on day 5 in parallel with myosin heavy chain (MyHC). siRNA-mediated MICAL1 silencing produced an ~1.7-fold accumulation of F-actin, while total β-actin protein remained unchanged, indicating a shift in the G-/F-actin equilibrium toward polymerization rather than altered actin expression. The accumulated F-actin reduced YAP1 phosphorylation, promoted its nuclear translocation, and increased the expression of the YAP1 target gene CTGF. MICAL1 depletion also enhanced myoblast proliferation: EdU incorporation and cell viability increased, and PCNA, CCNB1, and CCND1 protein expression was upregulated, while the cell cycle distribution shifted toward the G2/M phase, with a reciprocal loss in G0/G1. Concurrently, MICAL1 knockdown suppressed MyoD, Myogenin, and MyHC throughout differentiation and severely impaired myotube formation, with reductions in the fusion index, myotube area, and length. We conclude that MICAL1 is required for the proliferation-to-differentiation switch in myoblasts and that its activity restrains F-actin-driven YAP1 signaling to permit timely myogenic commitment. MICAL1 may therefore represent a candidate for further investigation in muscle-wasting diseases.

## 1. Introduction

Skeletal muscle accounts for roughly 50% of total body mass and is an essential organ that supports locomotion, respiratory mechanics, and systemic metabolic homeostasis [1]. The preservation of both muscle mass and functional integrity depends fundamentally on myogenesis, a highly ordered developmental and regenerative program activated in response to growth signals, mechanical overload, or pathological insult [2,3]. This program proceeds through sequential transitions: the proliferative expansion of muscle progenitor cells (myoblasts), their withdrawal from the cell cycle and commitment to the myogenic lineage, and the fusion of postmitotic myocytes into multinucleated contractile myotubes [4,5]. These transitions depend on coordinated cytoskeletal remodeling, activation of myogenic regulatory factors (MRFs), and suppression of mitogenic programs, all of which are required for proper muscle fiber maturation [6,7].

At the subcellular level, the actin cytoskeleton is a dynamic scaffold, and its precise remodeling is required for myogenesis [8]. Actin exists in two interconvertible forms: a soluble monomeric form (G-actin) and a polymerized filamentous form (F-actin). Continuous turnover between these pools, driven by ATP-dependent polymerization and depolymerization, sets the dynamic state of the cytoskeleton [9]. During differentiation, the actin network undergoes extensive reorganization to facilitate cell elongation, membrane reconfiguration, and the fusion events that generate syncytia [10,11]. This reorganization is controlled by a set of actin-binding proteins (ABPs) that mediate filament assembly, disassembly, branching, and stabilization [12,13]. Depletion of ABPs, including Twinfilin-1 [14] and Cofilin-2 [15], impairs myogenesis through mechanisms converging on the mechanosensitive transcriptional coactivator Yes-associated protein 1 (YAP1), establishing the actin–YAP1 axis as a critical node in myogenic fate decisions. However, those studies examined ABPs that bind actin filaments non-covalently. Enzymatic covalent modification of actin subunits is a distinct mode of cytoskeletal control, and whether actin-regulatory proteins of this kind also participate in myogenic regulation has not been examined.

MICAL1 (Molecule Interacting with CasL 1), a ~120 kDa flavoprotein monooxygenase originally identified as a semaphorin–plexin effector in repulsive axon guidance, comprises an N-terminal FAD-binding monooxygenase (MO) domain, a central calponin homology (CH) domain, and a C-terminal LIM domain [16]. The MO domain catalyzes the NADPH-dependent stereospecific oxidation of actin methionine residues Met44 and Met47, disrupting the hydrophobic inter-protomer contacts that stabilize the filament and thereby accelerating depolymerization [17,18]. Unlike non-covalent ABP-based or severing-based regulatory circuits, this enzymatic, covalent mechanism of actin control operates independently, adding a distinct tier of cytoskeletal regulation. MICAL1 has additionally been implicated in cytokinetic cortical actin remodeling, where it cooperates with Rab35 to drive oxidation-mediated F-actin disassembly required for ESCRT-III recruitment and abscission [19]. In skeletal muscle, MICAL1 transcripts are downregulated in starvation-induced myotube atrophy [20] and in tibial muscular dystrophy [21], implicating it as a protective factor whose loss may contribute to atrophic phenotypes. Despite the clinical relevance of sarcopenia and cachexia, muscle-wasting conditions characterized by defective myogenesis [3,22], the role of MICAL1 in skeletal myogenesis remains unexplored.

A central mechanism coupling the actin cytoskeletal state to cell fate is the mechanosensitive regulation of YAP1, the primary Hippo pathway effector [23,24]. The G-actin/F-actin ratio determines MST1/2–LATS1/2 kinase activity: elevated F-actin tension inhibits this cascade and prevents Ser127 phosphorylation of YAP1, releasing it from 14-3-3-mediated cytoplasmic retention [25,26]. Nuclear YAP1 then partners with TEAD transcription factors to induce pro-proliferative programs, including the upregulation of Cyclin D1 (*Ccnd1*) and the concomitant induction of cell cycle effectors, such as Cyclin B1 (*Ccnb1*) and the proliferation marker PCNA [27,28]. In the context of myogenesis, nuclear YAP1 maintains progenitor cells in a proliferative state [29,30] and directly antagonizes MyoD-dependent transcriptional activation, thereby preventing premature myogenic commitment [31,32,33]. Given the established role of MICAL1 in promoting actin depolymerization, we reasoned that MICAL1 might regulate the G-actin/F-actin balance during myogenic differentiation, with potential consequences for YAP1 mechanosensitive signaling.

In this study, we investigated whether MICAL1 contributes to myogenic differentiation in C2C12 progenitor cells and how its loss affects actin dynamics, YAP1 signaling, cell cycle progression, and the myogenic regulatory factor (MRF) cascade. siRNA-mediated knockdown of MICAL1 led to F-actin accumulation and nuclear localization of YAP1 in myoblasts. Cells depleted of MICAL1 proliferated more rapidly, with a selective expansion of the G2/M fraction. During differentiation, they failed to upregulate MyoD, Myogenin, or MyHC, with corresponding defects in myotube formation. These results indicate that MICAL1 is required to maintain F-actin homeostasis and YAP1-dependent control of myogenic progression, and they suggest that loss of MICAL1 may contribute to muscle-wasting pathology.

## 2. Results

### 2.1. Spatiotemporal Expression Profiling of MICAL1 During Myogenic Differentiation

**Figure 1:**
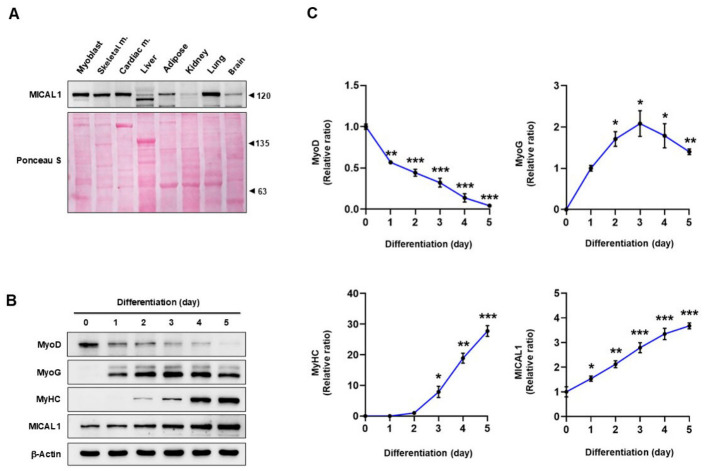
Characterization of MICAL1 expression profiles in murine tissues and throughout myogenic differentiation. (**A**) MICAL1 protein levels in C2C12 myoblasts and various tissues from 8-week-old mice. Ten micrograms of total protein were loaded per lane, and Ponceau S staining served as the loading control, as conventional housekeeping proteins (e.g., β-actin, α-tubulin) vary considerably between tissues. (**B**) Representative immunoblots of MRFs (MyoD, MyoG, and MyHC) and MICAL1 at the indicated time points during C2C12 differentiation. β-Actin served as the loading control. (**C**) Densitometric quantification normalized to β-Actin. Relative expression ratios were calculated against the following baseline time points: Day 0 for MyoD and MICAL1, Day 1 for MyoG, and Day 2 for MyHC. Data are presented as the mean ± SEM from three independent experiments (*n* = 3). Statistical significance was assessed by one-way ANOVA followed by Tukey’s multiple-comparisons test with asterisks denoting statistically significant differences (* *p* < 0.05, ** *p* < 0.01, *** *p* < 0.001).

Myoblast differentiation and fusion depend on precisely coordinated remodeling of the actin cytoskeleton [12,13]. Given the established role of MICAL1 as an enzymatic regulator of actin filament dynamics, we first characterized its endogenous expression across murine tissues and throughout the myogenic differentiation program. Immunoblotting, with Ponceau S staining as a loading control, showed that MICAL1 (~120 kDa) is broadly expressed across mouse tissues, with higher abundance in muscle-related tissues (myoblasts, skeletal muscle, and cardiac muscle) than in non-muscle tissues (liver, adipose, kidney, and brain). Among the non-muscle organs, the lung showed the highest MICAL1 expression, implying roles beyond contractile tissues (Figure 1A).

Longitudinal profiling across a 5-day differentiation period revealed characteristic expression dynamics for each myogenic marker (Figure 1B,C). MyoD declined progressively from day 0 onward, consistent with its role as a proliferative-phase commitment factor. Myogenin (MyoG) showed a transient peak on day 3, followed by a significant decline on day 5, reflecting its role in initiating but not sustaining terminal differentiation. MyHC accumulated steadily from day 2, reaching maximal expression by day 5. In contrast, MICAL1 increased progressively and significantly across the differentiation timeline, from day 1 to a sustained maximum on day 5, with a temporal trajectory close to that of MyHC. The upregulation of MICAL1 during terminal differentiation coincides with the extensive cytoskeletal reorganization required for myotube formation and membrane fusion. This temporal association is consistent with an active role in myogenic maturation, although functional perturbation will be needed to distinguish it from a permissive role.

### 2.2. MICAL1 Knockdown Potentiates F-Actin Accumulation and Promotes Nuclear Translocation of YAP1

**Figure 2:**
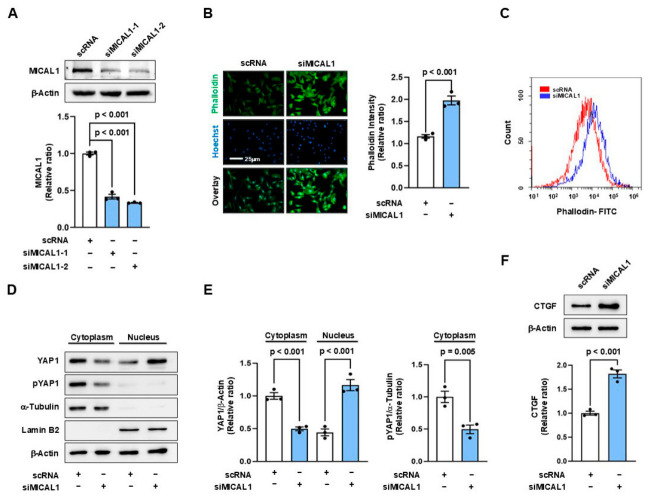
MICAL1 depletion facilitates F-actin accumulation and nuclear localization of YAP1. (**A**) Representative immunoblots and densitometric quantification of MICAL1 protein abundance in C2C12 myoblasts at 24 h post-transfection with scRNA, siMICAL1-1, or siMICAL1-2 (two independent siRNAs targeting distinct regions of Mical1, with comparable knockdown by both duplexes); expression levels were normalized to β-Actin, with the control set to unity. (**B**) F-actin architecture visualized by FITC-phalloidin staining (green) with nuclei counterstained with Hoechst 33342 (blue). Scale bar: 25 μm. FITC-Phalloidin intensities were quantified using ImageJ 1.54g software. (**C**) Flow cytometric assessment of F-actin levels in transfected myoblasts. (**D**) Subcellular fractionation and immunoblotting for YAP1 and pYAP1. α-Tubulin and Lamin B2 served as cytoplasmic and nuclear markers, respectively. (**E**) Densitometric quantification of cytoplasmic and nuclear YAP1 (normalized to β-Actin) and cytoplasmic pYAP1 (normalized to α-Tubulin), with relative ratios calculated against scRNA controls. (**F**) Immunoblotting for CTGF. Relative protein ratios were normalized to β-Actin. Data are presented as the mean ± SEM from three independent experiments (*n* = 3). Statistical significance was assessed by one-way ANOVA followed by Tukey’s multiple-comparisons test for panel (**A**) (three groups, namely scRNA, siMICAL1-1, and siMICAL1-2) and by unpaired two-tailed Student’s *t*-test for the two-group comparisons in panels (**B**,**C**,**E**,**F**). Exact *p*-values are indicated on the graphs (*p* < 0.05).

To investigate the functional role of MICAL1 in actin dynamics and downstream mechanosensitive signaling, we depleted MICAL1 in C2C12 myoblasts using two independent siRNAs targeting distinct regions of *Mical1* (siMICAL1-1 and siMICAL1-2; sequences listed in Table S1) [34]. Immunoblot analysis confirmed efficient knockdown by both duplexes, reducing MICAL1 protein to a comparable extent relative to scrambled control RNA (scRNA) at 24 h post-transfection in growth medium (Figure 2A). Because both siRNAs produced concordant effects, siMICAL1-1 (hereafter siMICAL1) was used for all subsequent experiments, and the principal phenotypes were independently confirmed with the second duplex, siMICAL1-2 (Figures S2–S4). FITC-Phalloidin staining demonstrated that MICAL1 depletion significantly increased F-actin levels by approximately 1.7-fold relative to scRNA controls (Figure 2B), corroborated by flow cytometric quantification showing a marked rightward shift in FITC-phalloidin fluorescence intensity (Figure 2C). By contrast, total β-actin protein was unchanged in siMICAL1-transfected cells as compared with scRNA controls in both whole-cell and fractionated lysates (Figure 2A,D). Because the phalloidin signal reports only the filamentous pool, whereas the immunoblot reports the entire β-actin pool, the selective rise in F-actin without a change in total β-actin indicates a shift in the G-/F-actin equilibrium toward polymerization rather than increased actin synthesis.

Subcellular fractionation, validated by α-Tubulin (cytoplasmic) and Lamin B2 (nuclear) markers (Figure 2D), revealed that MICAL1 silencing significantly reduced cytoplasmic YAP1 and cytoplasmic phospho-YAP1 (pYAP1)/α-Tubulin, with a concomitant and significant increase in nuclear YAP1 (Figure 2D,E). Total cellular YAP1 remained unchanged (Supplementary Figure S1), confirming that MICAL1 governs YAP1 phosphorylation status and nuclear availability without altering overall expression. The functional transcriptional activity of the nuclear YAP1 pool was directly confirmed by the drastic upregulation of CTGF protein, a canonical YAP1–TEAD target gene, in siMICAL1-transfected cells (Figure 2F). Importantly, the independent siMICAL1-2 duplex reproduced this YAP1 redistribution. Subcellular fractionation showed reduced cytoplasmic YAP1 and pYAP1, with a concomitant increase in nuclear YAP1, closely matching the siMICAL1 results (Supplementary Figure S2). The concordance between two non-overlapping siRNAs indicates that these effects reflect on-target MICAL1 depletion rather than off-target activity. MICAL1 is therefore required to maintain the actin polymerization state and the cytoplasmic retention of YAP1 in myoblasts.

### 2.3. MICAL1 Silencing Augments Myoblast Proliferation and Drives G2/M Cell Cycle Expansion

**Figure 3:**
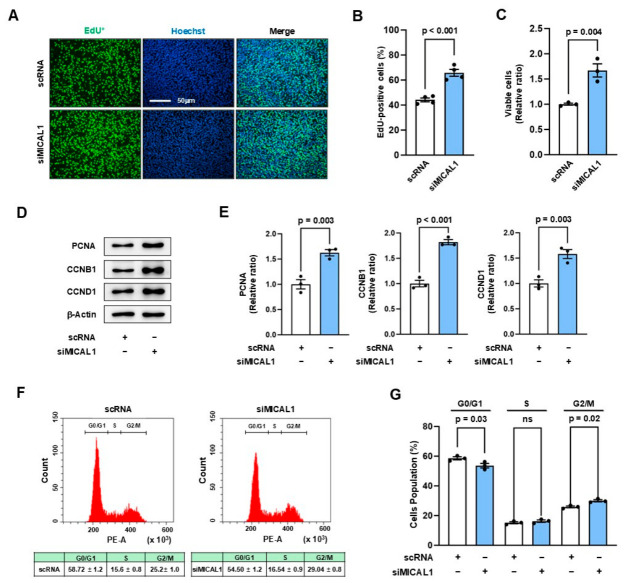
MICAL1 knockdown potentiates myoblast proliferative capacity. (**A**) Proliferation assays using EdU incorporation (green) and Hoechst 33342 counterstaining (blue). Scale bar: 50 μm. (**B**) Quantification of EdU-positive cells. (**C**) Viable cell counts determined by the WST-8 colorimetric assay. (**D**,**E**) Representative immunoblots showing protein expression of the canonical YAP1 target CCND1 together with the cell cycle effectors PCNA and CCNB1 in scRNA- and siMICAL1-transfected myoblasts; β-actin served as the loading control. (**F**,**G**) PI-stained flow cytometric cell cycle analysis: (**F**) representative DNA content histograms with inset mean ± SEM values for each phase, and (**G**) quantitative bar graph of phase distributions. Data are presented as the mean ± SEM from three independent experiments (*n* = 3). Statistical significance was assessed by unpaired two-tailed Student’s *t*-test for the two-group comparisons in panels (**B**,**C**,**E**) and for the comparison between the two siRNA conditions at each cell cycle phase in panel (**G**). Exact *p*-values are indicated on the graphs; statistical significance was set at *p* < 0.05. ns, not significant.

Nuclear YAP1 drives cell cycle entry and promotes proliferation through the transcriptional activation of pro-mitotic gene programs [28]. MICAL1 knockdown significantly increased the proportion of EdU-positive actively replicating cells (Figure 3A,B) and WST-8-assessed viable cell number (Figure 3C), indicating a hyper-proliferative state in siMICAL1-transfected myoblasts. Immunoblot analysis confirmed elevated protein expression of the canonical YAP1 target gene CCND1, accompanied by elevated expression of the proliferation marker PCNA and the mitotic cyclin CCNB1 (Figure 3D,E). PI-stained flow cytometric DNA content profiling revealed a selective and significant expansion of the G2/M-phase population (scRNA: 25.2 ± 1.0% vs. siMICAL1: 29.0 ± 0.8%) with a reciprocal contraction of the G0/G1 fraction (scRNA: 58.7 ± 1.2% vs. siMICAL1: 54.5 ± 1.2%), whereas the S-phase proportion was not significantly altered (Figure 3F,G). This selective G2/M accumulation is consistent with the pronounced induction of CCNB1, since the CCNB1/CDK1 complex serves as the principal driver of the G2-to-M transition, and indicates that MICAL1 depletion preferentially affects late cell cycle progression rather than uniformly accelerating the entire mitotic cycle. The pro-proliferative phenotype was independently reproduced by the second siRNA, siMICAL1-2. Relative to scRNA, siMICAL1-2 increased the viable cell number measured by a cell proliferation assay (Supplementary Figure S3). The concordance of two independent siRNAs targeting distinct regions of Mical1 indicates that the enhanced proliferation reflects on-target MICAL1 depletion rather than off-target activity.

### 2.4. MICAL1 Is Required for Myogenic Regulatory Factor Expression and Terminal Myotube Formation

Given that sustained cell cycle progression antagonizes myogenic commitment [4], we assessed whether MICAL1-mediated proliferative expansion impairs differentiation. C2C12 cells transfected with scRNA or siMICAL1 were subjected to a 5-day differentiation protocol, and MICAL1 and core MRF protein levels were monitored on days 0, 3, and 5.

**Figure 4:**
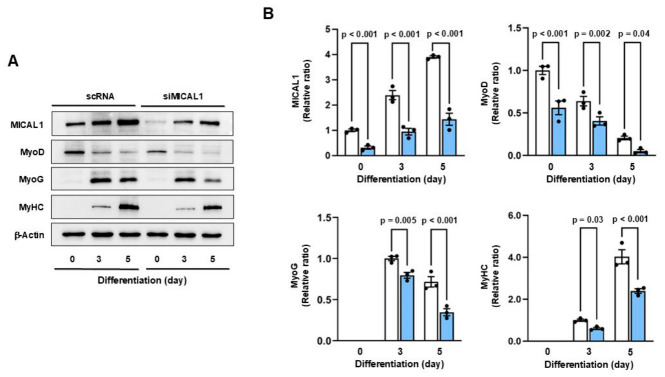
Loss of MICAL1 suppresses the induction of myogenic regulatory factors. (**A**) Immunoblots of MICAL1, MyoD, MyoG, and MyHC on differentiation days 0, 3, and 5 in scRNA- and siMICAL1-transfected C2C12 cells. β-Actin served as the loading control. (**B**) Densitometric quantification of protein abundance for scRNA (open columns) and siMICAL1 (filled columns). Expression was normalized to β-Actin, with ratios calculated against scRNA on day 0 (for MICAL1 and MyoD) or day 3 (for MyoG and MyHC). Data are presented as the mean ± SEM from three independent experiments (*n* = 3). Statistical significance was assessed by unpaired two-tailed Student’s *t*-test comparing the two siRNA conditions at each differentiation time point. Exact *p*-values are indicated on the graphs (*p* < 0.05). Figure 1 and Figure 4A derive from independent experiments imaged under different exposure conditions. Band intensities are therefore not compared across figures, and quantification was performed only within each experiment.

Immunoblot analysis confirmed persistent and significant MICAL1 protein reduction in siMICAL1 cells at all time points (Days 0, 3, and 5), validating sustained knockdown throughout the differentiation timeline (Figure 4A,B). In MICAL1-depleted cells, MyoD protein was significantly attenuated at all time points (Days 0, 3, and 5). Downstream MRFs were equivalently impaired: MyoG was significantly reduced on days 3 and 5, and MyHC was markedly diminished on days 3 and 5. Persistent suppression of MyoD, which directly induces MyoG expression to initiate terminal differentiation [35], indicates a commitment-level defect rather than a late-stage maturation failure.

**Figure 5:**
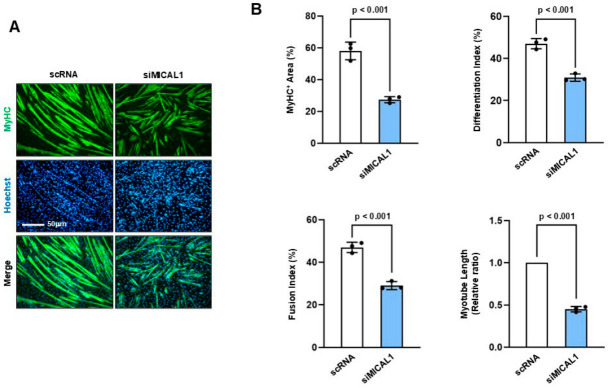
MICAL1 is required for multinucleated myotube formation. (**A**) Representative immunofluorescence images of differentiated C2C12 cells (Day 5) stained for MyHC (green, Alexa Fluor 488) and nuclei (blue, Hoechst 33342). Scale bar: 50 μm. (**B**) Quantitative morphometric analysis, including MyHC-positive area, differentiation index, fusion index, and myotube length. Data are presented as mean ± SEM from three independent experiments (*n* = 3). Statistical significance was assessed by unpaired two-tailed Student’s *t*-test. Exact *p*-values are indicated on the graphs (*p* < 0.05). These analyses were performed on differentiation day 5, when the cultures had reached confluence, so total nuclear number is comparable between conditions. The proliferative effect of MICAL1 depletion was evaluated separately under growth conditions in Figure 3.

Morphometric analysis of MyHC immunostaining at differentiation day 5 confirmed a severe failure of myotube formation: the MyHC-positive area was reduced from ~58% (scRNA) to ~27% (siMICAL1), the differentiation index fell from ~47% to ~30%, the fusion index declined from ~47% to ~28%, and myotube length was reduced to ~0.46-fold of the controls (Figure 5A,B). The independent siMICAL1-2 duplex produced the same differentiation defect. MyHC immunostaining on day 5 was markedly diminished, with corresponding reductions in MyHC-positive area, differentiation index, fusion index, and myotube length relative to scRNA (Supplementary Figure S4). Reproduction of the differentiation phenotype by a second, non-overlapping siRNA indicates that impaired myogenesis is a specific consequence of MICAL1 depletion rather than an off-target effect. Taken together, MICAL1 is required for both the transcriptional induction of the myogenic program and the morphological execution of myotube assembly.

## 3. Discussion

Our experiments place MICAL1 in the proliferation–differentiation switch of skeletal myogenesis. MICAL1 protein rose progressively during C2C12 differentiation in parallel with the terminal markers MyoG and MyHC. Knockdown of MICAL1 produced an aberrant accumulation of F-actin together with nuclear translocation of YAP1 and upregulation of the YAP1 target CTGF. YAP1 activation, in turn, supported myoblast proliferation, with a selective expansion of the G2/M fraction and increased expression of PCNA, CCNB1, and CCND1. The same hyper-proliferative state was accompanied by sustained suppression of MyoD, MyoG, and MyHC and by severe defects in myotube formation. To our knowledge, MICAL1 has not previously been examined as an actin-regulatory protein in skeletal muscle, and our results show that its loss in myoblasts disrupts the actin–YAP1 axis and impairs differentiation.

The progressive upregulation of MICAL1 during C2C12 differentiation, with expression remaining low in proliferating myoblasts and rising steadily to a maximum on day 5 in synchrony with MyHC (Figure 1), indicates that MICAL1 has an active role in terminal myogenic maturation. This temporal trajectory implies a progressively heightened demand for MICAL1-mediated actin depolymerization as cells exit the proliferative phase, precisely when the cytoskeletal state must shift from a proliferation-permissive, F-actin-rich configuration to a differentiation-permissive, G-actin-enriched state. The relatively high MICAL1 abundance in muscle-related tissues, including myoblasts and skeletal muscle, compared with non-muscle tissues (Figure 1A), is consistent with a sustained requirement for tightly controlled actin turnover in the myogenic lineage. This interpretation is reinforced by transcriptomic evidence from disease states: MICAL1 downregulation in starvation-induced myotube atrophy [20] and tibial muscular dystrophy [21] collectively implicates MICAL1 as a protective factor in muscle homeostasis. This differentiation-associated induction most likely accompanies the myogenic transcriptional program itself. As myogenic regulatory factors such as MyoD and MyoG activate the differentiation gene network, the demand for directed actin depolymerization rises during cell cycle exit, elongation, and fusion, and MICAL1 induction would support this transition. The precise upstream regulators driving MICAL1 induction during myogenesis were not examined here and remain to be defined.

The F-actin accumulation observed upon MICAL1 knockdown is consistent with the previously reported role of MICAL1 in promoting actin filament disassembly across diverse cellular contexts that require dynamic cytoskeletal remodeling [18,19]. The substantial elevation of F-actin upon MICAL1 depletion, together with unchanged total β-actin protein (Figure 2A,D), indicates that the phenotype arises from a shift in actin polymerization dynamics rather than from increased actin synthesis. Direct biochemical interrogation of MICAL1 catalytic activity in myoblasts was beyond the scope of this study and remains an important question for future investigation. We did not directly measure actin Met44/Met47 oxidation. The F-actin accumulation observed is consistent with reduced MICAL1-dependent depolymerization but does not, by itself, establish an oxidative mechanism. Consistent with a mechanosensitive function for MICAL1-dependent actin remodeling, MICAL1 was recently shown to mediate shear-activated, localized F-actin disassembly that supports mechanotransduction in platelets [36].

The F-actin accumulation in siMICAL1 myoblasts is associated with YAP1 nuclear translocation in a manner consistent with the canonical Hippo pathway, in which elevated cytoskeletal tension is known to suppress the MST1/2–LATS1/2 cascade, reduce Ser127 phosphorylation of YAP1, and release it from 14-3-3-mediated cytoplasmic retention [27,28,37]. Our fractionation data—significant cytoplasmic pYAP1 reduction, nuclear YAP1 enrichment, and upregulation of canonical YAP1 target genes, such as CTGF and CCND1 (Figure 2F and Figure 3E)—confirm that nuclear YAP1 is transcriptionally active. A comparable phenotype has been described in myoblasts depleted of CNN3, in which F-actin accumulation likewise drives YAP1 nuclear translocation and impaired differentiation [38]. This parallel supports a model in which nuclear YAP1 acts as an integrator of cytoskeletal imbalance in myogenic progenitors, such that disruption of actin homeostasis—regardless of the specific upstream regulator—must be resolved before differentiation can proceed. Whereas CNN3 and other previously characterized regulators of this axis act largely through structural or actin-binding mechanisms, MICAL1 is an actin-disassembling factor of a distinct molecular class, indicating that mechanistically different actin regulators can converge on this axis. The precise molecular basis of the effect of MICAL1 on F-actin in myoblasts, including any contribution of its enzymatic activity, was not examined here and remains to be defined. Together, these observations position MICAL1 upstream of the F-actin–YAP1 mechanotransduction axis in myoblasts. We did not assess the phosphorylation status of MST1/2 or LATS1/2, so the intermediate signaling between F-actin accumulation and reduced YAP1 phosphorylation were not directly established in this system. F-actin can influence YAP1 both through LATS-dependent phosphorylation and LATS-independent mechanisms, and distinguishing between these routes will require further investigation.

Nuclear enrichment of YAP1 in siMICAL1 cells drives a pro-proliferative transcriptional program, as evidenced by enhanced EdU incorporation and increased viable cell counts. The modest but significant G2/M expansion, in the absence of a significant S-phase shift, is consistent with the pronounced elevation of CCNB1, since the CCNB1/CDK1 complex drives the G2-to-M transition and is induced downstream of YAP1–TEAD activity [39]. MICAL1 has also been reported to participate in cytokinetic cortical actin remodeling [19]. However, we did not observe cytokinesis failure or multinucleation, and our data do not address whether cytokinesis is affected in myoblasts. Beyond driving proliferation, nuclear YAP1 has been shown to antagonize MyoD-dependent transcriptional activation in skeletal muscle progenitors [31,32,33]. This dual pro-mitotic and anti-myogenic function of nuclear YAP1 plausibly accounts for the sustained MyoD suppression observed throughout the differentiation timeline in siMICAL1 cells (Figure 4). MICAL-family proteins have been implicated in proliferation control in other settings, although the direction of the effect appears to be context-dependent. Depletion of MICAL1 or MICAL2 suppresses proliferation in several cancer cell types [40,41], whereas in myoblasts, MICAL1 loss enhances proliferation, underscoring cell-type-specific functions.

The sustained MyoD suppression in siMICAL1 cells identifies a primary commitment-level defect in the myogenic cascade. Because MyoD directly occupies and activates the *Myogenin* promoter and other late MRF target elements [35], its persistent attenuation from day 0 onward provides a mechanistic explanation for the downstream suppression of MyoG (days 3 and 5) and MyHC (days 3 and 5). The reduction in MyoD in proliferating (day 0) myoblasts already indicates that MICAL1 loss compromises myogenic commitment during the proliferative phase, consistent with elevated nuclear YAP1 antagonizing MyoD before differentiation is induced, rather than a defect confined to later stages. These molecular failures culminate in the severe morphometric deficits observed on day 5: reduced MyHC-positive area, differentiation index, fusion index, and myotube length (Figure 5). The fusion failure in siMICAL1 cells reflects the transcriptional commitment defect attributable to MyoD suppression. Although controlled, transient actin polymerization is itself required for myoblast fusion [10,11], the sustained and global F-actin accumulation caused by MICAL1 loss during the proliferative phase is distinct from the locally regulated actin dynamics that drive fusion. The defect we observe is therefore positioned upstream, at the level of myogenic commitment, so that MICAL1-depleted cells do not reach the fusion-competent state. Several questions raised by these findings invite further investigation, including the upstream signals driving MICAL1 upregulation during myogenic differentiation, the in vivo relevance of MICAL1 in muscle-specific conditional knockout and injury-regeneration models, and the integration of MICAL1-mediated actin regulation with extracellular mechanical cues encountered during muscle regeneration.

Reduced *Mical1* transcripts in muscle-atrophy datasets [20,21], together with the differentiation defects we observed after MICAL1 knockdown, are consistent with a contribution of MICAL1 loss to impaired regenerative capacity in muscle disease. Whether restoring or maintaining MICAL1 expression has therapeutic value in sarcopenia, cachexia, or muscular dystrophy will need to be tested in vivo.

Several limitations of this study should be noted. First, although our conclusions derive from loss-of-function, potential off-target effects were addressed by using two independent siRNAs targeting distinct regions of *Mical1*. Both reduced MICAL1 expression and reproduced the principal phenotypes, namely nuclear YAP1 enrichment with reduced pYAP1 (Supplementary Figure S2), enhanced proliferation (Supplementary Figure S3), and impaired myogenic differentiation (Supplementary Figure S4), and the scrambled control was matched for double-stranded RNA load. A titratable or siRNA-resistant re-expression system would nonetheless be required to establish sufficiency formally. A formal test of the requirement for YAP1 in this pathway would similarly need controlled, partial modulation of YAP1 activity rather than complete ablation because YAP1 is itself required for normal myogenesis, and this is an important extension of the present work. Second, the increase in filamentous actin was assessed by phalloidin staining and flow cytometry, which report relative F-actin rather than the absolute G-/F-actin ratio, so biochemical G-/F-actin fractionation would provide a more direct measure. Third, we did not measure the expression of the related family members MICAL2 and MICAL3 because all three share F-actin-depolymerizing activity, any compensatory upregulation would be expected to counteract, rather than produce, the F-actin accumulation we observed, whereas the absence of compensation would indicate a non-redundant role for MICAL1. Finally, the functional analyses rely on the immortalized C2C12 cell line. However, C2C12 is a well-established model of myogenic differentiation, validation in primary myoblasts or satellite cells, as well as in muscle-injury-regeneration or muscle-specific Mical1 conditional-knockout models, will be required before extending these conclusions to muscle physiology or disease.

## 4. Materials and Methods

### 4.1. Murine Tissue Acquisition and Processing

Tissue samples were obtained from 8-week-old male C57BL/6 mice that had been acclimated under standard laboratory conditions and euthanized by cervical dislocation. All in vivo experimental protocols were strictly conducted in accordance with the guidelines and received prior approval from the Institutional Animal Care and Use Committee (IACUC) of Dongguk University (Approval No. IACUC-2021-009). Following excision, tissues were immediately rinsed with ice-cold physiological saline to remove residual blood and processed using a mechanical tissue homogenizer (Omni International, Inc., Kennesaw, GA, USA). For protein expression profiling, 10 μg of total protein from each tissue lysate was subjected to immunoblot analysis with a specific anti-MICAL1 antibody (Table S2).

### 4.2. Cell Culture and Myogenic Differentiation

C2C12 murine myoblasts (CRL-1772; ATCC, Manassas, VA, USA) were maintained in growth medium (GM) consisting of Dulbecco’s Modified Eagle Medium (DMEM; Gibco, Carlsbad, CA, USA) supplemented with 10% fetal bovine serum (FBS) and 100 units/mL penicillin/streptomycin (Gibco). To initiate the myogenic program, myoblasts were seeded in 35 mm culture dishes at an initial density of approximately 1.3 × 10^5^ cells per dish. Upon reaching ~90% confluency, differentiation was induced by transitioning to differentiation medium (DM) composed of DMEM supplemented with 2% horse serum (Gibco). Cells were cultured in DM for up to 5 days, with the medium replenished every 24 h.

### 4.3. siRNA-Mediated Gene Silencing

Myoblasts were seeded at 1.3 × 10^5^ cells per 35 mm dish and cultured for 20–24 h until reaching 40–50% confluency. Cells were transfected with 200 nM of MICAL1-specific siRNA (siMICAL1-1; Bioneer, Daejeon, Republic of Korea) or siMICAL1-2, or with non-targeting scrambled control RNA (scRNA; Genolution, Seoul, Republic of Korea) using Lipofectamine 2000 (Invitrogen, Waltham, MA, USA) in serum-free DMEM for 4 h. siMICAL1-1 (sense 5′-CAGGUGCCAUGACUAAGUAUU-3′) targets mouse *Mical1* (NM_138315) and corresponds to the previously published and experimentally validated MICAL1-silencing sequence [34]; siMICAL1-2 is an independently designed duplex targeting a non-overlapping region of *Mical1*. Unless otherwise indicated, siMICAL1-1 is hereafter referred to as siMICAL1 and was used for all experiments, with siMICAL1-2 used to confirm key phenotypes, and the same 200 nM concentration was applied to the scRNA control so that the non-specific double-stranded RNA load was matched between groups. Cells were then transitioned to GM for 24 h prior to harvest or subsequent assays. Specific oligonucleotide sequences are detailed in Table S1.

### 4.4. Subcellular Fractionation

Cytoplasmic and nuclear fractions were isolated using NE-PER Nuclear and Cytoplasmic Extraction Reagents (Thermo Fisher Scientific, Waltham, MA, USA) at 24 h post-transfection. Harvested cell pellets were incubated on ice with Cytoplasmic Extraction Reagent I (CER I) for 30 min, followed by the addition of CER II. After centrifugation at 15,000 rpm for 15 min at 4 °C, the supernatant (cytoplasmic fraction) was collected. The remaining pellet was resuspended in Nuclear Extraction Reagent (NER) to yield the nuclear fraction. Equal protein aliquots from both fractions were equilibrated prior to immunoblotting. α-Tubulin and Lamin B2 were used as cytoplasmic and nuclear fraction markers, respectively.

### 4.5. Immunoblotting

Cellular proteins were extracted using lysis buffer (PBS containing 2% Triton X-100, 0.2 mM PMSF, and 1% phosphatase inhibitor cocktail II; Sigma-Aldrich, St. Louis, MO, USA). Protein concentrations were determined by the Bradford assay. Equal protein samples (20 μg) were resolved by SDS-PAGE and transferred onto nitrocellulose membranes (Amersham Biosciences, Piscataway, NJ, USA). Membranes were blocked with 5% non-fat dry milk in TBST (TBS with 0.5% Tween-20) and incubated overnight at 4 °C with primary antibodies. Following washing, membranes were incubated with HRP-conjugated secondary antibodies (1:10,000) and visualized using TOPview™ ECL Femto Western Substrate (Enzynomics, Daejeon, Republic of Korea). For comparison of MICAL1 expression across different mouse tissues (Figure 1A), equal amounts of total protein (10 μg per lane) were loaded, and Ponceau S staining of the membrane was used as the loading control instead of conventional housekeeping proteins since β-actin, α-tubulin, and GAPDH expression varies substantially among tissue types. Band densities were quantified using Fusion Solo imaging software (Evolution-Capt v17.04). Detailed antibody information is provided in Table S2.

### 4.6. Immunocytochemistry and Morphometric Analysis

Cells were fixed with 4% paraformaldehyde, permeabilized with 0.3% Triton X-100, and blocked with 3% bovine serum albumin (BSA). Samples were incubated overnight at 4 °C with anti-Myosin Heavy Chain (MyHC) antibody (1:100), followed by an Alexa Fluor 488-conjugated secondary antibody (Invitrogen) and counterstaining with Hoechst 33342. Fluorescent images were captured across five randomly selected fields using a Leica fluorescence microscope (Mannheim, Germany). Myogenic indices—including differentiation index, fusion index, and myotube length—were quantified using ImageJ 1.54g software as previously described [14]. The differentiation index was calculated as the ratio of MyHC-positive nuclei within myotubes to the total number of nuclei. The fusion index was defined as the percentage of myotubes containing three or more nuclei relative to the total number of nuclei counted. F-actin architecture was visualized using FITC-conjugated phalloidin (Cytoskeleton, Inc.) and quantified using ImageJ.

### 4.7. Cell Proliferation and Viability Assays

For proliferation analysis, transfected cells were incubated with 10 μM EdU (5-ethynyl-2′-deoxyuridine) for 4 h at 37 °C using the Click-iT™ EdU Kit (Invitrogen). EdU-positive nuclei were detected via Click-iT reaction chemistry and visualized alongside Hoechst-stained total nuclei using a Leica fluorescence microscope; quantification was performed in five randomly selected fields per condition using ImageJ. Cell viability was assessed 24 h post-transfection using the WST-8-based Quanti-Max Cell Viability Assay Kit (BioMax, Seoul, Republic of Korea), with absorbance measured at 450 nm using a microplate reader (Model 680; Bio-Rad, Hercules, CA, USA).

### 4.8. Flow Cytometry

For cell cycle profiling, harvested myoblasts were fixed in 70% ethanol overnight at 4 °C. Fixed cells were treated with RNase A (100 µg/mL, 30 min, 37 °C), stained with propidium iodide (PI; 50 µg/mL; Abcam, Cambridge, UK) for 20 min in the dark, and analyzed on a CytoFLEX platform (Beckman Coulter, Brea, CA, USA). Doublets and debris were excluded on FSC-A/FSC-H and PI-A/PI-W plots, and DNA content histograms of single cells were modeled to determine the G0/G1, S, and G2/M fractions. For F-actin quantification, harvested cells were fixed with 4% paraformaldehyde at room temperature for 20 min, permeabilized with 0.1% Triton X-100, stained with FITC-conjugated phalloidin (Cytoskeleton, Inc., Denver, CO, USA), and fluorescence intensity distributions were compared between scRNA- and siMICAL1-transfected cells.

### 4.9. Statistical Analysis

All experimental data are presented as the mean ± standard error of the mean (SEM) from a minimum of three independent biological replicates (*n* ≥ 3). Statistical significance was evaluated using one-way analysis of variance (ANOVA) followed by Tukey’s post hoc test for multiple comparisons, or by an unpaired two-tailed Student’s *t*-test for two-group comparisons. Comparisons between the two siRNA conditions at each differentiation time point and at each cell cycle phase were made using unpaired two-tailed Student’s *t*-tests. Normality was assessed using the Shapiro–Wilk test prior to parametric analysis, and the specific test applied is indicated in each figure legend. A *p*-value < 0.05 was considered statistically significant. Statistical analyses were performed using GraphPad Prism 8.0.2 (Boston, MA, USA).

## 5. Conclusions

In summary, MICAL1 is required for the orderly switch from proliferation to differentiation in C2C12 myoblasts. Upon MICAL1 depletion, F-actin accumulates, YAP1 translocates into the nucleus, and the canonical YAP1 target CCND1 is upregulated together with the downstream cell cycle effectors CCNB1 and PCNA and the YAP1–TEAD readout CTGF. These transcriptional changes are accompanied by enhanced proliferation, with a selective expansion of the G2/M-phase population, while MyoD, MyoG, and MyHC remain suppressed throughout the differentiation timeline, ultimately leading to failed myotube formation. Together, these findings implicate the actin–YAP1 axis as a key contributor to the differentiation defect that follows MICAL1 loss. Whether MICAL1 or this signaling axis can be leveraged therapeutically in conditions of impaired muscle regeneration—such as sarcopenia, cachexia, or muscular dystrophy—will require validation in vivo and disease-relevant models.

## Data Availability

The original contributions presented in this study are included in the article/Supplementary Materials. Further inquiries can be directed to the corresponding author(s).

## Supplementary Materials

The following supporting information can be downloaded at https://www.mdpi.com/article/10.3390/ijms27146505/s1.
