# Supplementary material for: MICAL1 Contributes to Myogenic Differentiation by Modulating Actin Remodeling and YAP1 Nuclear Localization in C2C12 Myoblasts
Source: Int J Mol Sci. 2026 Jul 22;27(14):6505. doi: 10.3390/ijms27146505 (PMC13409775; doi:10.3390/ijms27146505)
Supplement: Supplementary file 1 [file ijms-27-06505-s001.zip › Supplement Figure S1-S4.pdf]

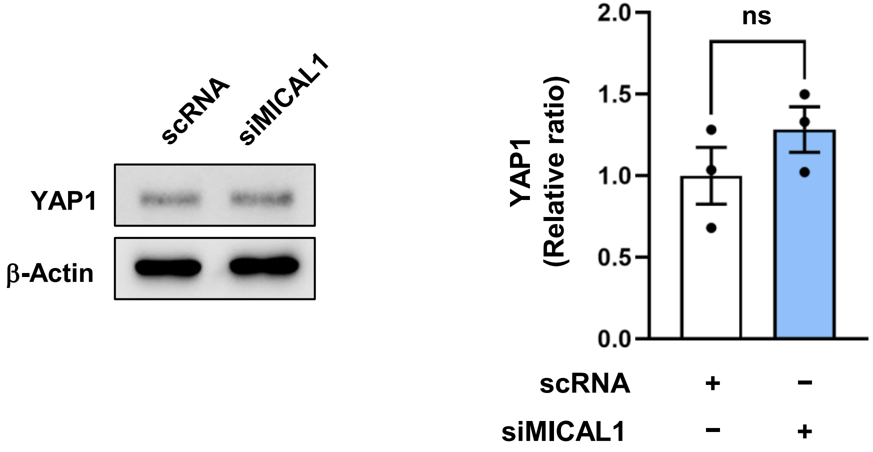

Supplementary Figure S1. Total cellular YAP1 is unchanged after MICAL1 knockdown. C2C12 myoblasts were transfected with scrambled control RNA (scRNA) or siMICAL1 (siMICAL1-1) and harvested under growth conditions. Whole-cell lysates were immunoblotted for total YAP1 with β-Actin as a loading control. The bar graph shows total YAP1 normalized to β-Actin (relative ratio). Data are mean ± SEM (n = 3); unpaired two-tailed Student's t-test; ns, not significant.

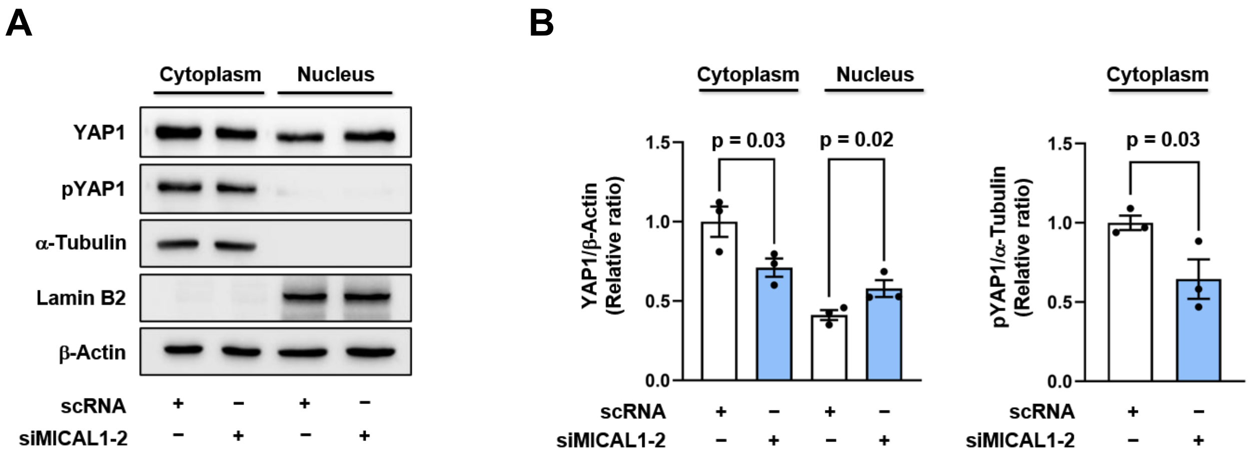

Supplementary Figure S2. An independent second siRNA (siMICAL1-2) reproduces the redistribution of YAP1. (**A**) C2C12 myoblasts transfected with scRNA or siMICAL1-2 were fractionated into cytoplasmic and nuclear compartments and immunoblotted for YAP1 and pYAP1; α-Tubulin and Lamin B2 served as cytoplasmic and nuclear markers, respectively, and β-Actin as a loading reference. (**B**) Quantification of cytoplasmic pYAP1 (normalized to α-Tubulin) and of cytoplasmic and nuclear YAP1 (normalized to β-Actin), expressed as relative ratios. Data are mean ± SEM ($n = 3$); unpaired two-tailed Student's t-test ($p = 0.02$, 0.03, and 0.03 for the three quantified comparisons).

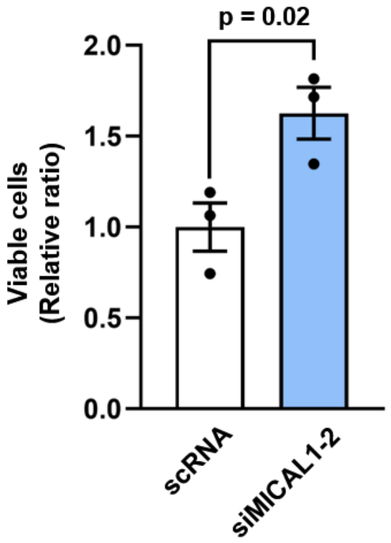

Supplementary Figure S3. An independent second siRNA (siMICAL1-2) reproduces the pro-proliferative phenotype. Viable cell number was measured by a cell proliferation assay in C2C12 myoblasts transfected with scRNA or siMICAL1-2 and is expressed as a relative ratio. Data are mean ± SEM (n = 3); unpaired two-tailed Student's t-test ($p = 0.02$).

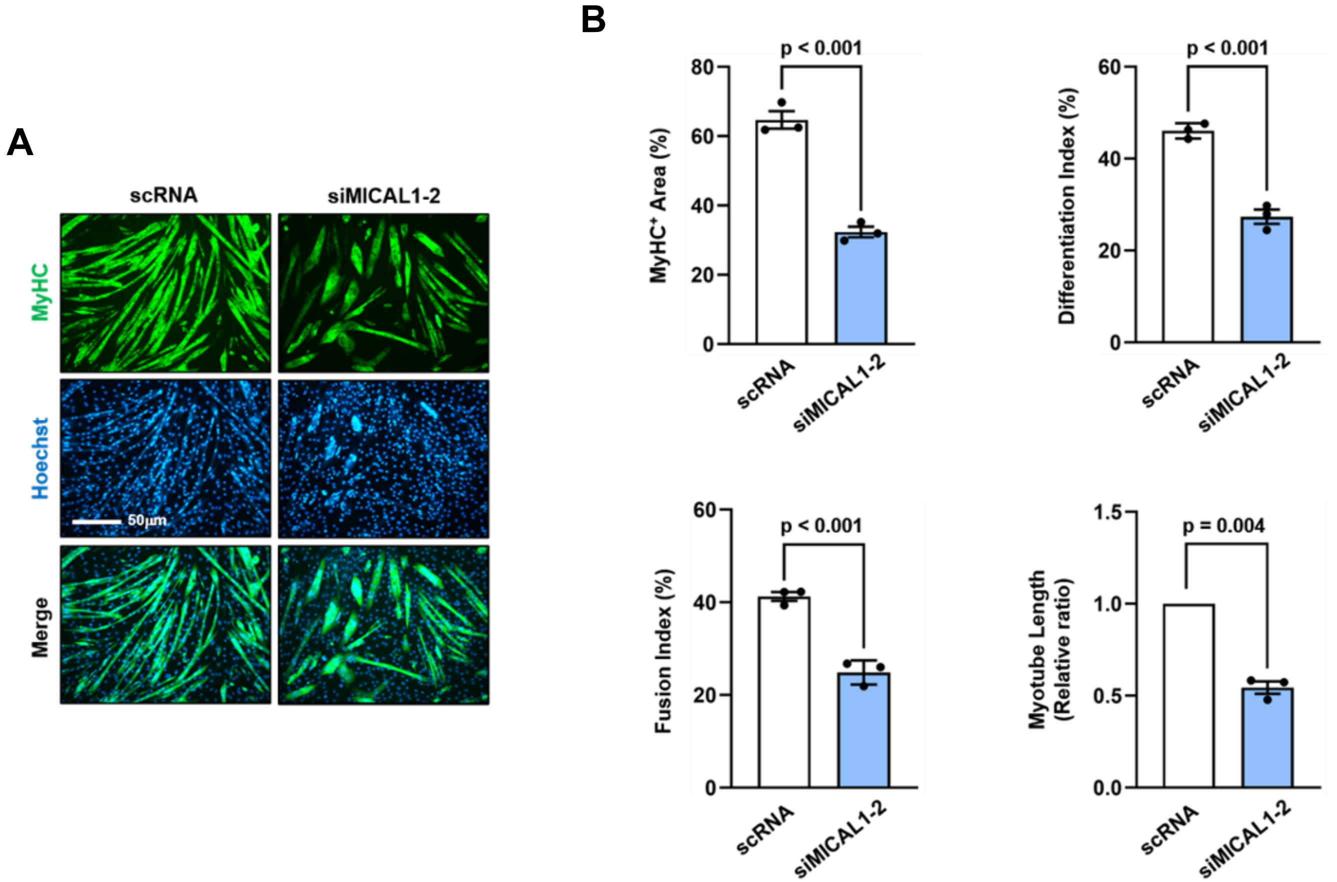

Supplementary Figure S4. An independent second siRNA (siMICAL1-2) impairs myotube formation. (**A**) Representative immunofluorescence images of C2C12 cells transfected with scRNA or siMICAL1-2 and differentiated for 5 days, stained for MyHC (green, Alexa Fluor 488) and nuclei (Hoechst 33342, blue), with merged images shown. Scale bar, 50 μm. (**B**) Quantitative morphometric analysis of MyHC-positive area, differentiation index, fusion index, and myotube length. Data are mean ± SEM ($n = 3$); unpaired two-tailed Student's t-test (MyHC-positive area, differentiation index, and fusion index, $p < 0.001$; myotube length, $p = 0.004$).
