# Supplementary material for: MICAL1 Contributes to Myogenic Differentiation by Modulating Actin Remodeling and YAP1 Nuclear Localization in C2C12 Myoblasts
Source: Int J Mol Sci. 2026 Jul 22;27(14):6505. doi: 10.3390/ijms27146505 (PMC13409775; doi:10.3390/ijms27146505)
Supplement: Supplementary file 1 [file ijms-27-06505-s001.zip › Supplementary Table S1,2.pdf]

Table S1. Oligonucleotide sequences for transfection

Gene	Oligonucleotide sequence (5´-3´)
scRNA (control RNA)	UCACAACCUCCUAGAAAGAGUAGA
siMICAL1-1	CAGGUGCCAUGACUAAGUAUU
siMICAL1-2	CGUAAGCAAGACACCAUCA

Table S2. Antibodies list

Antibody	Type	Targeted species	Manufacturer	Cat. No.	Dilution ratio*
MICAL1	Polyclonal	Rabbit	Proteintech, Rosemont, Illinois, USA	14818-1-AP	1:3,000
MyHC	Monoclonal	Mouse	DSHB, Iowa, IA, USA	MF20	1:10,000
MyoD	Monoclonal	Mouse	Santa Cruz Biotechnology, Dallas, TX, USA	sc-377460	1:1,000
MyoG	Monoclonal	Mouse	Santa Cruz Biotechnology, Dallas, TX, USA	sc-12732	1:1,000
YAP1	Monoclonal	Rabbit	Cell Signaling Technology, Danvers, MA, USA	14074S	1:10,000
pYAP1	Polyclonal	Rabbit	Cell Signaling Technology, Danvers, MA, USA	4911S	1:10,000
Lamin B2	Monoclonal	Rabbit	Abcam, Cambridge, United Kingdom	ab151735	1:10,000
α-Tubulin	Monoclonal	Mouse	DSHB, Iowa, IA, USA	12G10	1:10,000
CCNB1	Polyclonal	Rabbit	Proteintech, Rosemont, Illinois, USA	55004-1-AP	1:3,000
CCND1	Monoclonal	Mouse	Proteintech, Rosemont, Illinois, USA	60186-1-lg	1:10,000
PCNA	Polyclonal	Rabbit	Proteintech, Rosemont, Illinois, USA	10205-2-AP	1:10,000
CTGF	Polyclonal	Rabbit	Proteintech, Rosemont, Illinois, USA	25474-1-AP	1:5,000
β-Actin	Monoclonal	Rabbit	Sigma-Aldrich Chemical, St. Louis, USA	A2066	1:10,000
Antibodies HRP-linked anti-rabbit IgG			Cell Signaling Technology, Danvers, MA, USA	#7074	1:10,000
Goat anti-mouse(H+L)			Thermofisher Sci., Waltham, MA, USA	#32430	1:2,000

*All blots were visualized using a TOPview ECL Femto (Enzynomics).
